# Single cell ecology

**DOI:** 10.1098/rstb.2019.0076

**Published:** 2019-10-07

**Authors:** Thomas A. Richards, Ramon Massana, Stefano Pagliara, Neil Hall

**Affiliations:** 1 Biosciences and Living Systems Institute, University of Exeter, Stocker Road, Exeter EX4 4QD, UK; 2 Department of Marine Biology and Oceanography, Institut de Ciències del Mar (CSIC), 08003 Barcelona, Spain; 3 Earlham Institute, Norwich Research Park, Norwich, NR4 7UZ, UK; 4 School of Biological Sciences, University of East Anglia, Norwich, NR4 7TU, UK

**Keywords:** microbe, interactions, methodology, symbiosis, protist, Archaea

## Abstract

Cells are the building blocks of life, from single-celled microbes through to multi-cellular organisms. To understand a multitude of biological processes we need to understand how cells behave, how they interact with each other and how they respond to their environment. The use of new methodologies is changing the way we study cells allowing us to study them on minute scales and in unprecedented detail. These same methods are allowing researchers to begin to sample the vast diversity of microbes that dominate natural environments. The aim of this special issue is to bring together research and perspectives on the application of new approaches to understand the biological properties of cells, including how they interact with other biological entities.

This article is part of a discussion meeting issue ‘Single cell ecology’.

## 1. Introduction

The study of biological processes often relies on ensemble measurements for the purpose of capturing and comparing sample means. Yet the specific circumstance of each data point, or each individual organism, which make up any distribution used to calculate a mean and characterize a phenomenon is hugely important. Therefore, being unable to separate and interrogate the nature of each individual entity within a distribution of data has hampered our ability to understand many biological phenomena. For single-celled organisms, determining the specific circumstance of the individuals within a population is key for understanding: (i) the variation upon which selection can act to result in evolutionary adaptation [1,2], and (ii) the diversity of rare forms present in a natural environment which can rise to prominence in the event of environmental perturbation [3]. This is a hindrance when we consider the fact that much of the interesting biology we seek to understand occurs on the scale of a single cell, a scale that is often difficult to study experimentally.

Consider for example a simple experiment to ascertain the susceptibility of a clonal population of bacteria to antibiotic treatment. A comparison of an antibiotic-treated population versus an untreated population with a subsequent measurement of the mean number of surviving cells can give an idea of the overall efficacy of the antibiotic in use. However, it has been shown within clonal populations of bacteria that there can be considerable differences in physiological functions between individual cells [4–6]. Specifically, within the treated population reside bacterial cells that can survive such treatment, persisting until the drug is removed, when a subpopulation composed of what is known as persister cells resume cell division [7,8] and a second subpopulation known as viable but non-culturable cells remain dormant [9]. Our simple experiment to ascertain the efficacy of the antibiotic will fail to capture these clinically relevant subpopulations thus leading to a false estimate of the capability of the antibiotic in use to clear bacterial infections. Noteworthy, a similar heterogeneity in phenotypic responses to environmental stressors has been observed in a variety of species across the tree of life [10–12]. To understand the persister cell outcome we must study the individual circumstance of the cells that make up the population of study. We must understand each individual's cellular history, the nature of its transcriptome, its physiology, and describe the heterogeneity across the population. We must also understand how individual cells interact with their environment and with each other. It is clear from persister and viable but non-culturable cell studies that microbial cell heterogeneity can be relevant for understanding human health outcomes and, indeed, single cell approaches, specifically those based on the use of microfluidic technologies [13–15], have been used to explore numerous features of these phenomena [16–23].

The persister and viable but non-culturable cell examples illustrate how single cell approaches can be used to explore a biological phenomenon in extensive detail. Interestingly, it also hints at the complexity of the dimensions of study that is accessible with the application of single cell methodologies. If a single clonal lineage [17] of bacteria can show physiological heterogeneity underpinning survival beyond antibiotic challenge in laboratory culture, the complexity of cellular phenotypes which operate in the multiple dimensions present in natural communities and across diverse ecosystems must be considerable. The development of technologies to isolate, manipulate and analyse single cells offers an opportunity to explore this complexity. The aim of this issue is to present scientific progress and emerging questions relating to the use of single cell technologies to answer a diversity of biological questions. In the past our inability to both separate and integrate molecular/cellular/physical processes at the single cell level has meant that our understanding of biological systems has been necessarily superficial. The ability to separate, recover, perturb and measure traits of individual cells in order to understand the biological genotypes and phenotypes on the scale of a single cell is changing biological sciences. This change promises a route into understanding the individual variations and circumstances that underpin many key patterns (or ‘distributions’) important for understanding biological phenomena.

## 2. A working definition for ‘single cell ecology’ for the purpose of this issue

This issue, and the associated scientific meeting, brings together a diversity of scientists from disparate disciplines who are advancing both research and technological approaches to understand biology on the level of a single cell. This is a necessarily loose association of scientific enterprise and as such a broad definition for the term ‘single cell ecology’ is useful here. We therefore define the theme single cell ecology as: ‘the use of state-of-the-art approaches, often informed by physical and molecular methods, to study biological phenomena at the scale of a single cell with a focus on how individuals or groups of individuals of the same species interact with their environment, each other and cells of different species.’ We are aware of the oxymoron in the title because of course one cannot study ecology at the scale of a single individual, but it is the implementation of single cell methods to explore ecological interactions that we are particularly interested in developing here. Indeed, many of the papers in this issue engage with new approaches and perspectives on microbial interactions which have become accessible through the use of single cell methods.

The single cell methods we are interested in exploring in this issue cover a wide field of investigation. Previously published key examples of progress in this field have focused on the study of: heterogeneity in antibiotic responses within populations of cells [10,11,17], how cancer cell lines have evolved [24], how transcriptome expression profiles vary across cells during viral infection [25], detailed study of the cell cycle [26], physical properties of a cell [27] and how uncultivated microbes interact with each other and their environment [28]. This science has in part been driven by the application of new methods in physics for fluid manipulation [13,14] and the diverse use of fluorescent activated cell sorting [29–31]. Such approaches, combined with methodological leaps in molecular biology and bioinformatics [32–34], have enabled the sequencing of ‘whole’ genomes and transcriptomes from single cell samples [31,35–39], which has opened up numerous fields of novel investigation. This is revolutionizing how we study disease, but many of the methods developed in the application of this technology to medical sciences are now being applied to the study of microbial ecology and exploration of the microbial tree of life. As such, the single cell approach is allowing researchers, for the first time, to study the genomes, cellular properties and perhaps even behavioural phenotypes of the uncultured microbial forms that occupy most ecosystems [36,40–43].

## 3. Aims of this issue

This themed issue brings together a range of disparate scientific fields including researchers using physics-informed methodology to manipulate cells to understand their physical and chemical properties; molecular biologists developing methods for sampling nucleotides, proteins and metabolites from single cells; evolutionary biologists who are trying to sample microbes directly from environments and understand where they branch on the tree of life; genome biologists who are trying to understand gene function, the wider diversity of gene repertoires and the ancestry of gene families; and microbial ecologists who seek to understand microbial functions and interactions in natural environments. The range of contributors have been chosen with the aim of showcasing how this technology has opened the door to new insights. However, there is a bias in the constellation of work included as we have purposefully sought to try and demonstrate how many of the techniques and approaches that were pioneered initially with a medical focus have now been repurposed for understanding the ecology and evolutionary context of microbes. We also note that in several cases this repurposing is only in its infancy and as such many of the articles represent ‘a call to arms’ with regard to further development. Therefore, there is a slight dichotomy in the selection of papers. Some of them present work that is only just starting ‘to get a grip’ on the vast diversity and function of unknown microbes present in natural environments. This is contrasted by more definitive application of single cell approaches for studying known cellular systems and microbial phenomena at new levels of detail in synthetic environments (the study of persister cells mentioned above is a prime example). Many early fields of endeavour are characterized by similar dichotomies as researchers apply new methods to a diversity of established questions.

To broadly summarize, the objectives of this issue are to: (i) cover and promote the diversity of single cell technological approaches for studying biological phenomena; (ii) identify successful and powerful applications of these approaches for understanding microbial ecological systems; and (iii) allow researchers to identify new ways to combine well-established and novel approaches and bring them to fundamental research questions. In several articles such progress is clearly set out providing examples of how synergistic approaches have led to substantial progress.

## 4. Overview of the contributions

We have divided the papers into four broad themes, although in many cases the papers occupy more than one of these categories as highlighted in the introduction to these four themes below. Again, we hope that this will demonstrate how synergistic this work can be and how this work can lead to new perspectives and progress. In many cases this work focuses on tackling the study of microbial interactions in natural environments. The four themes are loosely as follows: (a) progress in methodological and technological development but with a focus on what additional development is needed and where application of single cell approaches could be beneficial; (b) how single cell approaches have led to significant advances in understanding the properties and functions of cells as individuals and cells interacting with their environment and community; (c) how single cell approaches have been applied to the study of the uncultured microbial ‘world’ that represent the majority of microbial forms [44] in natural environments; and (d) how single cell approaches are revealing new information with regards to ecological interactions.

### (a) Methodological and technological developments and future challenges

This category includes work that makes use of technical progress in the manipulation of cells allowing them to be recovered, studied or exposed to chemical, physical or biological stimuli in a controlled and measurable manner. Work reported here includes articles that showcase approaches focusing on microfluidic systems that can be used to manipulate cells [45], or the growth of a population of cells [46,47] so they can be studied in detail. Microfluidic devices are essentially tiny plumbing systems, with dimensions ranging from one millimetre down to one micrometre, that is a hundred times smaller than the diameter of a human hair. Remarkably, these devices allow the manipulation of both individual cells [48] and the fluidic environment around them [13,14,49]. These approaches are allowing researchers to bend, stretch, compress, feed, drug, and image microbes in new ways (e.g. [27,45,48,50–54]). Variants of such apparatus also allow for the study of growth in isolation, growth under stimuli and growth in a community [55–58]. These papers describe application of single cell technologies for studying known phenomenon in unprecedented detail, often where understanding the heterogeneity of cellular systems/states is key for understanding the biological phenomenon of interest. One feature that is striking about these approaches is the extent of replication in these experiments, from hundreds to millions of individuals studied per experiment, which is allowing new levels of scientific rigour in the study of phenotypic characteristics that can be highly cryptic on the scale of a cell (e.g. [27,45,48,50,58]). Excitingly, such approaches are also now being developed for the study of environmental microbes (e.g. [25,37]), opening up the possibility of studying ‘wild’ microbes in a similar detail to what can be achieved for the study of laboratory cultured cells.

The second area covered in this category, which we wanted to highlight here, because the further development of this technology will enhance many fields of single cell biology, is the application of improved single cell transcriptome approaches. Such approaches require the development of methodologies for sampling ‘omics material for sequencing from tiny quantities of template (DNA for genome sequencing and RNA for transcriptome sequencing). A number of such approaches have been developed, as reviewed by Ku & Sebé-Pedrós with a focus on microbial eukaryotes. Their paper highlights how, as these technologies move out of infancy, this field promises to reveal much about gene expression signatures of distinct cellular physiological states and the transcriptional dynamics of microbial interactions [59]. The paper focuses on microbial eukaryotes, yet it is important to mention that much of this work has a foundation in medical sciences, and indeed single cell methods, specifically transcriptome methods, have recently underpinned progress in both understanding infection biology (e.g. the application of these technologies to understand transcriptome dynamics of parasite infection) and tissue development in human cells (e.g. [60–66]). These works, and the associated approaches, are important milestones in the development of the human and parasite cell atlas projects [67,68]. A related and notable step forward, for example, has been the ability to sequence in parallel both DNA and RNA material from a single cell [69], a methodology which, with further development, we predict will be very important for environmental microbiology research. Such approaches have only become viable with the development of second and third generation sequencing technologies, but numerous limitations are still evident in such approaches specifically associated with: recovery of ‘omic templates (i.e. mRNA), template amplification, short read sequencing technologies and the validity of subsequent ‘omic assemblies. Because of this we feel it is important to highlight progress in moving towards enhanced transcriptome sampling methods combining long read technologies and new bioinformatic approaches specifically seeking to address error correction and the production and assembly of transcriptome data. Progress in this area is reviewed by Bryne and colleagues [70].

The special issue also includes multiple articles highlighting current progress and areas of limitation in a particular field of biological or ecological research. These articles call for further development of interdisciplinary single cell approaches that could potentially lead to progress in these fields. For example, Sebastián and Gasol highlight the need for further development of cell visualization approaches or the combination of established visualization approaches with newly developed molecular methods for studying microbes in ocean samples and other natural environments [71]. They argue that such progress is necessary for understanding the relationship between specific microbes, their activity/function and their growth.

Santoro *et al*. highlight the need for further work in the study of Archaea from the environment, discussing how many natural populations of Archaea are under-sampled and understudied [72]. Their article discusses how single cell methods have led to some progress but highlights the need for further methodological development specifically for targeted or enriched recovery of Archaea for cellular manipulation, culturing or molecular analysis. The paper also states the merit of going back to ‘classical’ culture-based approaches for microbial research, a theme highlighted elsewhere in this issue [73].

Similarly, Keeling discusses the gap between the under-representation of protist sampling and the extensive diversity of these groups in most natural environments, but makes a specific call for future work to seek to combine the promise of single cell ‘omic approaches with assessment of cellular structure, morphology and microbial behaviour [73]. Keeling argues that protists represent their own unique problem, because many of the key traits of protist taxa are impossible to evaluate directly from genome data. For example, swimming behaviours cannot be identified based on the repertoire of genes identified in a genome. This is an important consideration given the emerging extent of uncultured protist diversity [74–77]. Consequently, without an appropriate understanding of these cellular/behavioural contexts, exploration of the evolutionary and ecological nature of cryptic protist taxa would remain impoverished. This fits into the wider theme also touched on by Santoro *et al*. and Sebastián and Gasol [71,72], of the need to link up phenotypic data acquisition and other contextual data retrieval with targeted ‘omic sampling. Indeed, the development of methods that allow combined recovery of phenotypic data and material for ‘omics analysis could also drive further improvements for the targeted recovery of microbes that display specific behaviour or cellular characteristics, a subject that is raised throughout the issue (e.g. [78]). Keeling therefore calls for efforts in this direction and mentions the need for additional method development, suggesting a possible important role for microfluidic approaches in sampling protists.

### (b) Understanding the properties and functions of cells as individuals and components of an environment

The issue also includes several papers that make use of single cell approaches to study properties and functions of cells as individuals and/or components of an environment. Again, some of these papers focus on the use of microfluidic devices. One research paper reported here demonstrates how such devices can be used to study how microbial cell populations can collectively function to exchange metabolites and resist antibiotic treatment [46]. Another example of microfluidic methodologies featured here allowed Łapińska and co-authors to explore a new perspective on bacterial ageing, demonstrating that inheritance of maternal versus daughter cellular poles at cell division determines the rate of cellular division in the progeny cells [47]. This illustrates how single cell approaches are providing new perspectives on fundamental biological process like ageing and cell division (see also [26,79]).

With a focus on human heart muscle cells, Pires and colleagues demonstrate the use of microfluidic systems to assess the real-time deformability of a single cell, that is, how squashable a cell is, and demonstrate that this parameter is important for understanding the maturation state of heart muscle cells [45]. These authors also studied the effect of pharmacological treatment on this cellular property. This approach, which has also been used to understand and identify cellular properties of stem and blood cells [27,50], could have a vast array of applications for assaying cellular states in a number of different cell types, including microbial forms isolated directly from natural environments.

On a different scale and for a different purpose, several papers reported here discuss the utility of detecting cellular characteristics for targeted cell sorting from environmental samples. This has included, for example, the use of fluorescent activated cell sorting (FACS) approaches to sort and recover cells of specific size and with the presence/absence of photosynthetic pigments such as chlorophyll from environmental samples. These methods have also been combined with the use of dyes that label acidic vacuoles as a proxy for predatory-phagotrophic function (the ability of one cell to swallow another cell and digest it) in free-living protists [31,78,80]. These approaches have been linked up with protocols for single cell or small-scale metagenome (mini-metagenomes) sequencing, an approach that aims to sequence all the DNA material within a sample, in order to recover genome samples from uncultured microbes [29,31,39,80–82]. Several of the papers collected here make use of data retrieved from such approaches [83–85], which allowed recovery of previously unsampled taxa (discussed further in §4c).

One of the most challenging groups to classify, sample and study are the predatory mixotrophs: eukaryotic microbes that function as both photosynthetic algae and phagotrophic consumers of other cells. These cells occupy multiple levels in food webs and their function is important for many ecosystems [86]. Wilken and colleagues review the status of exploration of these groups, discussing the opportunity and limitation of multiple single cell approaches [78]. The challenge of studying this lifestyle suffers from many of the limitations outlined by Keeling [73]. This is because such groups are hard to consistently sample, and the mixotrophic behaviour, specifically phagotrophy, can be difficult to observe within a small photosynthetic cell. Furthermore, cellular and genomic markers for such behaviour are ill-defined both in terms of cellular staining protocols and the lack of identifiable proteome characteristics uniquely responsible for phagocytosis [87].

### (c) The study of uncultured microbes

The application of molecular methods to reconstruct a ribosomal RNA (rRNA) gene phylogeny, pioneered by Woese and colleagues [88,89], revolutionized our understanding of the tree of life. Based on approaches derived from this work, Pace [90] and others (e.g. [91–96]) developed methods for sampling rRNA gene sequences directly from environmental DNA, allowing the characterization of microbial community diversity directly from environmental samples. Such approaches were then updated with high-throughput parallel sequencing methods [97–99]. These protocols removed the necessity to culture microbes from the process of defining microbial diversity in natural environments and therefore eliminated a huge source of bias [94,100,101]. Collectively, these steps forward changed our understanding of microbial biodiversity, demonstrating a huge range of microbial forms many of which can occupy pivotal and uncharted sections of the tree of life [41,102]. However, such approaches offer only a snap-shot of biodiversity because they only sample a single semi-conserved gene marker, which has limitations for phylogenetic analysis [103] and tells us little about the characteristic of the cellular source other than that the cell sampled possesses a ribosome.

Metagenomic approaches have further expanded this picture and have allowed us to identify additional branches on the tree of life and importantly marry small subunit (SSU) rRNA gene sequences (used initially to characterize microbial diversity) to assembled, or partially assembled, genomes [29]. Such approaches have been particularly successful for archaeal and bacterial diversity (e.g. [41,104]). However, these methods often produce incomplete genome assemblies and many subsections of the microbial community investigated remain under-sampled. Furthermore, metagenomic approaches are limited for sampling microbial eukaryotes because eukaryotes have complex genome architectures composed of large portions of non-coding genome, intron/exon gene structures, variant GC biases across a chromosome and linear chromosome structures, meaning they are difficult to assemble from metagenome sequence datasets. However, the smallest eukaryotic cells can sometimes represent promising candidates for these metagenomic studies given their small and compact genomes [42,105] which can allow for improved genomic assembly.

Single cell technologies allow the further refinement of these metagenomic approaches, permitting the recovery of ‘omic material directly from a single cellular—granular—unit [29,36,37,40,41]. However, in practice (as discussed in §4d) such sampling often recovers a multi-unit microbiome within the single propagule sampled. Independent of the ‘purity’ of the biological entities sampled, single cell ‘omics approaches require template amplification [106], which often leads to both bias and partial recovery of the ‘omic material. Across the literature, there has been a disparate collection of single cell genomic methods applied to eukaryotes [36,82,107], all of which revealed that the genomic sampling recovered from such approaches is partial. A possible development in this area is the use of a co-assembly of the sequencing data from separate cells belonging to the same population [39]. Although this can miss single cell variability, this approach can recover near complete *de novo* genomes for uncultured lineages, representing a consensus gene inventory. So, a pertinent question is, in the absence of further methodological refinement allowing for consistent and complete genome recovery from a single cell sample, what are such datasets also good for?

As demonstrated in this issue, single cell genomic approaches can provide an effective means of sampling fragments of genome from uncultured cells that branch in intermediate and previously unsampled positions in the tree of life, lineages previously only known from environmental SSU rRNA gene sampling. For example, López-Escardó *et al*. provide a case study where the use of single cell genomic sampling allowed recovery of genome sample datasets from four divergent choanoflagellates (the protistan group that branches sister to the animal radiation [108]) from marine waters [84]. These four protists occupy phylogenetic branches not previously sampled using ‘omics methods [109]. The subsequent data were used to re-evaluate the evolutionary ancestry of multiple gene families implicated in the evolution of animal multicellular systems, providing evidence that additional examples of gene families that encode these pathways arose before the origin of the animals.

Wideman *et al*. also report a highly fragmented single cell genome sequence of a kinetoplastid-like cell, again from a marine environment [85]. The kinetoplastid group includes numerous animal parasites [110], and the cell recovered here represents a highly divergent relative to parasites that cause sleeping sickness in humans. Kinetoplastids and their sister clade, the diplonemids, have two different and highly unusual mitochondrial gene/genome structures [111]. Wideman and colleagues used the partial genome data recovered to further investigate the evolutionary history of these characteristics [85], filling in some gaps in the evolution of these divergent mitochondrial genomes and demonstrating that partial genomes from single cell methods can be useful for reconstructing the evolution of divergent cellular characteristics.

An additional use of the partial repertoire of genes recovered from single cell sequencing approaches is the identification of a collection of gene sequences for the purpose of multi-gene phylogeny, which can allow for improved phylogenetic resolution (e.g. [112,113]). Galindo and colleagues provide a case study of such an approach focusing on sampling of nucleariid amoebae [114], a protist group that is known to branch close to the primary radiation of the fungi [115]. Interestingly, in this case study the authors could directly tie their genome sampling to information about behaviour, morphology and ecology of the target protists. This approach allowed the authors to use their amended phylogenetic framework to reconstruct some biological characteristics of the last common ancestor of the Holomycota, the progenitor of the anciently derived clade that includes the fungi and the nucleariids. This provides an exemplar case of how improved phylogenetic resolution combined with single cell genomics and phenotypic data can be used to improve our understanding of ancient evolutionary transitions. Importantly, this paper includes comparisons of single cell genome, single cell transcriptome and culture based ‘omic approaches in order to compare efficacy of the recovery of a serviceable set of phylogenetic markers. The work demonstrates a minimal recovery of target genes compared to traditional culture-based transcriptome sequencing; however, the data recovered using the single cell approaches proved tractable for placing the target taxa within a phylogenetic tree. This identifies a possible route forward for resolving many more under-sampled and unresolved areas of the tree of life as single cell ‘omic approaches can provide enough data to allow for improved phylogenetic reconstruction. However, we note that in a few contentious areas of the tree, often characterized by poorly defined phylogenetic signatures, phylogenetic resolution may require a larger-scale of gene sequence sampling than is currently serviceable using single cell methods.

### (d) How are single cell approaches revealing new information regarding ecological interactions

One noticeable trend evident in this issue and in the field generally is a move towards understanding the complexity of interactions on the micro scale. Much of this work is reliant on the use of single cell approaches. As mentioned above, Dal Co and colleagues describe the use of microfluidic approaches to investigate how interacting bacterial cells generate metabolic gradients through the uptake and release of metabolites, a property that supports cross feeding behaviour [46]. Excitingly the work reported includes analysis of individual cellular growth rates as well as the expression of key metabolic genes within these structured populations, which proved vital for understanding both cross feeding behaviour and how elements of the experimental population can resist antibiotic treatment.

In a study that also sought to explore interactions within bacterial populations, Ebrahimi *et al*. used a combination of computational modelling and culture-based experiments to investigate the consequences of differing extracellular enzymatic secretion functions in multi-cellular aggregates of different strains of bacteria [116]. Remarkably, this work demonstrates that different secretion behaviours were directly linked to alternative multi-cellular aggregate strategies/phenotypes in marine bacteria.

As discussed above, protist single cell genome datasets recovered directly from environmental samples are presented in many articles in this issue. An additional use of such data, on top of those mentioned in §4c, includes the possibility of using ‘single’ cell datasets for exploring interactions represented within the ‘omics datasets recovered. This is an attractive prospect because many protists exhibit a complex range of interactions with other microbes (e.g. [117]). Indeed, it is precisely because protists form complex interactions that they are often difficult to grow in the laboratory; they are reliant upon a microbial interaction which is cryptic and/or difficult to propagate in sustainable culture. It is becoming increasingly clear that DNA and RNA profiles recovered from ‘single-cell’ propagules often have mixed provenance, representing both the putative ‘host’ cell, its associated microbiome (including viral infections), its prey (e.g. [80,82]) and indeed nucleotides that have contaminated it from the associated environment. Study of the mixed provenance of these ‘omic sequences can therefore be used to explore microbial interactions. Tyml *et al*. [118] sets out the progress and use of such approaches and summarizes how nine previously published single cell genomic datasets have provided evidence of single cell protist–microbiome interactions, including associations with viruses and putative intracellular bacteria.

The articles presented in this issue provide several cases of additional single cell data. In three papers, evidence for microbial interactions captured in sequence datasets are presented. These include two papers detailing evidence of a viral–host relationship [83,119] and a putative association between a protist and a candidate endosymbiotic bacterium [114]. Tyml and colleagues set out the challenge for the further development of these methods for distinguishing the nature of the ecological circumstance that have resulted in the mixed microbial signals identified in single cell ‘omic datasets [118]. Do these mixed signatures represent food, friend (mutualist), infection (parasitism) or contamination? Related to this call for further method development, Tyml and colleagues make the case for the need for large-scale datasets and an associated database to facilitate the study of single cell interactions, perhaps a single cell variant of similar efforts focused on cataloguing the diversity of symbioses [120].

The progression of the microbe–microbe interaction research theme, and indeed many of the themes of this issue, ultimately rests on the need to develop new methods. Florenza *et al*. grasp this challenge head on and discuss in detail the tailoring and application of emulsion, paired isolation and concatenated PCR (epicPCR) [121] for large-scale sampling of co-association between protists and associated prokaryotes [122]. This represents an exciting prospect that can potentially allow the study of co-associations on a huge scale, a scale tractable for valid statistical analysis, and removes the requirement for expensive nucleotide amplification and sequencing processes that would be required for single cell ‘omic approaches.

## 5. Conclusion and outlook

The papers covered in this issue demonstrate a diversity of research fields all making progress on developing approaches to manipulate, sample and experiment with single cells. The research papers, reviews and opinion pieces cover a range of important applications for these technologies discussing subjects such as the physical manipulation of cells, sampling of ‘omics data, understanding of pathogen–host life cycle, how microbes interact through their ‘feeding’ processes, how microbes can mediate against antibiotic challenge, and identifying and exploring interactions among uncultured microbial forms.

This issue identifies areas of research where progress has been considerable but also where further development could revolutionize future progress. Key examples include: (i) the need for systems to allow the link up of improved imaging and phenotype assays with ‘omic recovery from individual cells in environmental samples. Such approaches are needed to provide contextual data and also to allow for improved targeted recovery of cells within uncultured lineages. Linked to these suggestions there is a clear need for continued effort to expand and possibly automate high throughput systems for microbial culture from natural environments. (ii) The need for improved approaches for combined ‘omic sampling from single cell analysis (e.g. methods that allow the simultaneous recovery of DNA, mRNA and small RNA molecules). This work should be combined with the further development of long read sequencing technologies for use on small quantities of input template. (iii) Combined with these efforts we also need new methods for improved classification of mixed provenance ‘omic signatures as symbiotic, prey or contamination, allowing us to deconvolve mixed molecular signatures into microbial interactions. In all these cases we see vast opportunities for further technological development rooted in the use of tools developed within the field of physics, in particular using microfluidic platforms. Key examples of such technologies and approaches are showcased here. We look forward to the further development of this field and the further insights that will be provided.

## Data accessibility

This article has no additional data.
